# Identification of methylation-driven genes related to the prognosis of papillary renal cell carcinoma: a study based on The Cancer Genome Atlas

**DOI:** 10.1186/s12935-020-01331-7

**Published:** 2020-06-12

**Authors:** Zeyu Liu, Yuxiang Wan, Ming Yang, Xuewei Qi, Zhenzhen Dong, Jinchang Huang, Jingnan Xu

**Affiliations:** 1 Third Affiliated Hospital, Beijing University of Chinese Medicine, Beijing, 100029 China; 2 School of Traditional Chinese Medicine, Beijing University of Chinese Medicine, Beijing, 100029 China

**Keywords:** Papillary renal cell carcinoma, Methylation-driven genes, TCGA, Biomarkers

## Abstract

**Background:**

Aberrant DNA methylation patterns are involved in the pathogenesis of papillary renal cell carcinoma (pRCC). This study aimed to investigate the potential of methylation-driven genes as biomarkers in determining the prognosis of pRCC by bioinformatics analysis.

**Methods:**

DNA methylation and transcriptome profiling data were downloaded from The Cancer Genome Atlas database. Methylation-driven genes (MDGs) were obtained using MethylMix R package. A Cox regression model was used to screen for pRCC prognosis-related MDGs, and a linear risk model based on MDG methylation profiles was constructed. A combined methylation and gene expression survival analysis was performed to further explore the prognostic value of MDGs independently.

**Results:**

A total of 31 MDGs were obtained. Univariate and multivariate Cox regression analysis identified eight genes (CASP1, CD68, HOXD3, HHLA2, HOXD9, HOXA10-AS, TMEM71, and PLA2G16), which were used to construct a predictive model associated with overall survival in pRCC patients. Combined DNA methylation and gene expression survival analysis revealed that C19orf33, GGT6, GIPC2, HHLA2, HOXD3, HSD17B14, PLA2G16, and TMEM71 were significantly associated with patients’ survival.

**Conclusion:**

Through the analysis of MDGs in pRCC, this study identified potential biomarkers for precision treatment and prognosis prediction, and provided the basis for future research into the molecular mechanism of pRCC.

## Background

Renal cell carcinoma (RCC), a common malignant tumor in the urinary system, accounts for 2–3% of human cancers [1]. Papillary renal cell carcinoma (pRCC) represents the second most common pathological subtype of RCC, accounting for 18.5% [2, 3]. According to morphological characteristics, pRCC is usually divided into two main subtypes: type 1 and type 2 [4]. Studies have proved that type 2 pRCC tumors are more aggressive and have a worse prognosis than clear cell renal cell carcinoma (ccRCC) [5]. Compared with ccRCC patients, patients with metastatic pRCC usually have a worse prognosis [6]. Currently, pathological and clinical grading and staging still act as the main prognostic factor [7]. In recent years, with the development of molecular biology technology and bioinformatics, new biomarkers have the potential to be used as prognostic factors for different types of cancer, including pRCC. For instance, some studies have found that CYP4A11 expression is a potential poor prognostic factor for RCC [8], while others have detected long non-coding RNAs that might serve as prognostic biomarkers for pRCC [9], and some alternative splicing events also correlate with pRCC prognosis [10]. Therefore, finding new molecular markers to predict the clinical outcome of pRCC is of great significance for understanding pRCC’s molecular pathological characteristics, prognosis, and treatment.

Epigenetic modification plays an essential role in the development of human cancer [11]. Methylation, one of the most important epigenetic modifications, is involved in many cellular processes, including cell differentiation, genome stability, and gene imprinting [12], and it intimately relates with tumorigenesis [13]. Changes in DNA methylation can provide important evidence for the early diagnosis and prognosis of cancer, and offer new ideas for further clinical applications. Some previous studies have explored the relationships between methylation-driven genes and the prognosis of certain cancers, such as esophageal squamous cell carcinoma [14], lung adenocarcinoma [15], and bladder cancer [16], but currently, there is no such report for pRCC.

The Cancer Genome Atlas (TCGA) is an open-source database on cancer genes [17], from which researchers around the world can obtain and analyze relevant data. MethylMix, an algorithm based on R programming [18, 19], can identify disease-specific hypermethylated and hypomethylated genes. In this study, we extracted methylation and RNA expression data of pRCC patients from the TCGA database, obtained the methylation-driven genes related to pRCC using the MethylMix algorithm, and then constructed a Cox survival prediction model to evaluate the prognosis of pRCC, aiming at finding independent prognostic biomarkers, and exploring the correlation between abnormal DNA methylation and pRCC genome level to provide the scientific basis for personalized diagnosis and treatment.

## Methods

### Data acquisition and analysis

We downloaded the methylation data and transcriptomics data from KIRPs in the TCGA database. Methylation data were obtained from 276 tumor samples and 45 adjacent non-tumor samples from the Illumina Human Methylation 450 k platform; transcriptome profile data were from 289 tumor samples and 32 adjacent non-tumor samples without isoform expression and miRNA expression quantification. First, we normalized the downloaded data and analyzed the differences using the limma package of R software [20] to obtain differentially expressed genes (DEGs) and differentially methylated genes (DMGs), and then used the MethylMix package for integrated analysis. MethylMix is a statistical package of R software that can find methylation-driven genes by integrating DNA methylation data and RNA expression data. Using the MethylMix algorithm, we calculated the correlation between the gene methylation level and gene expression and obtained the methylation-driven genes after constructing a β-mixed model with a filtration of|logFC| > 0.5, P < 0.05, and Cor < − 0.3. TCGA data are publicly available without permission from the ethics committee.

### Functional enrichment, pathway analysis, and genetic alteration analysis

After obtaining the methylation-driver genes, to further understand the biological functions of these selected genes, we conducted Gene Ontology (GO) analysis and Kyoto Encyclopedia of Genes and Genomes (KEGG) pathway enrichment analysis [21] using the clusterprofiler package of R software [22]. GO analysis includes the molecular function (MF), biological process (BP), and cellular component (CC) [23]. We set P < 0.05 as the cutoff and visualized the results using Goplot [24]. The cBio Cancer Genomics Portal (cBioPortal) is an essential online platform for analyzing cancer genomics data [25]. We used cBioPortal to investigate the genetic alterations of methylation-driven genes in pRCC patients (TCGA, PanCancer Atlas).

### Prognostic model construction and survival analysis

To analyze the relationship between methylation-driven genes and prognosis, we conducted a survival analysis based on these methylation-driven genes via the survival package after combining the clinical data and prognosis of pRCC in TCGA. Only patients with complete survival information were included. We screened the prognosis-related methylation-driven genes with P < 0.05 and constructed a predictive model by multivariate Cox regression analysis. The risk score of each patient was calculated based on the model. The patients were separated into high-risk and low-risk groups after obtaining the median risk score, and the time-dependent receiver operating characteristic (ROC) curve was used for testing. The Kaplan–Meier survival curve was used to evaluate the overall survival rate of patients in high and low-risk groups. The log-rank test was used to determine whether the overall survival rate differed between the high-risk group and the low-risk group. P < 0.05 was considered statistically significant. In addition, we performed joint survival analysis of the methylation-driven gene level and gene expression level in pRCC patients to further identify key genes related to prognosis, and obtain the patient’s joint survival curve via the survival R package.

## Results

### Data acquisition and analysis

**Fig. 1:**
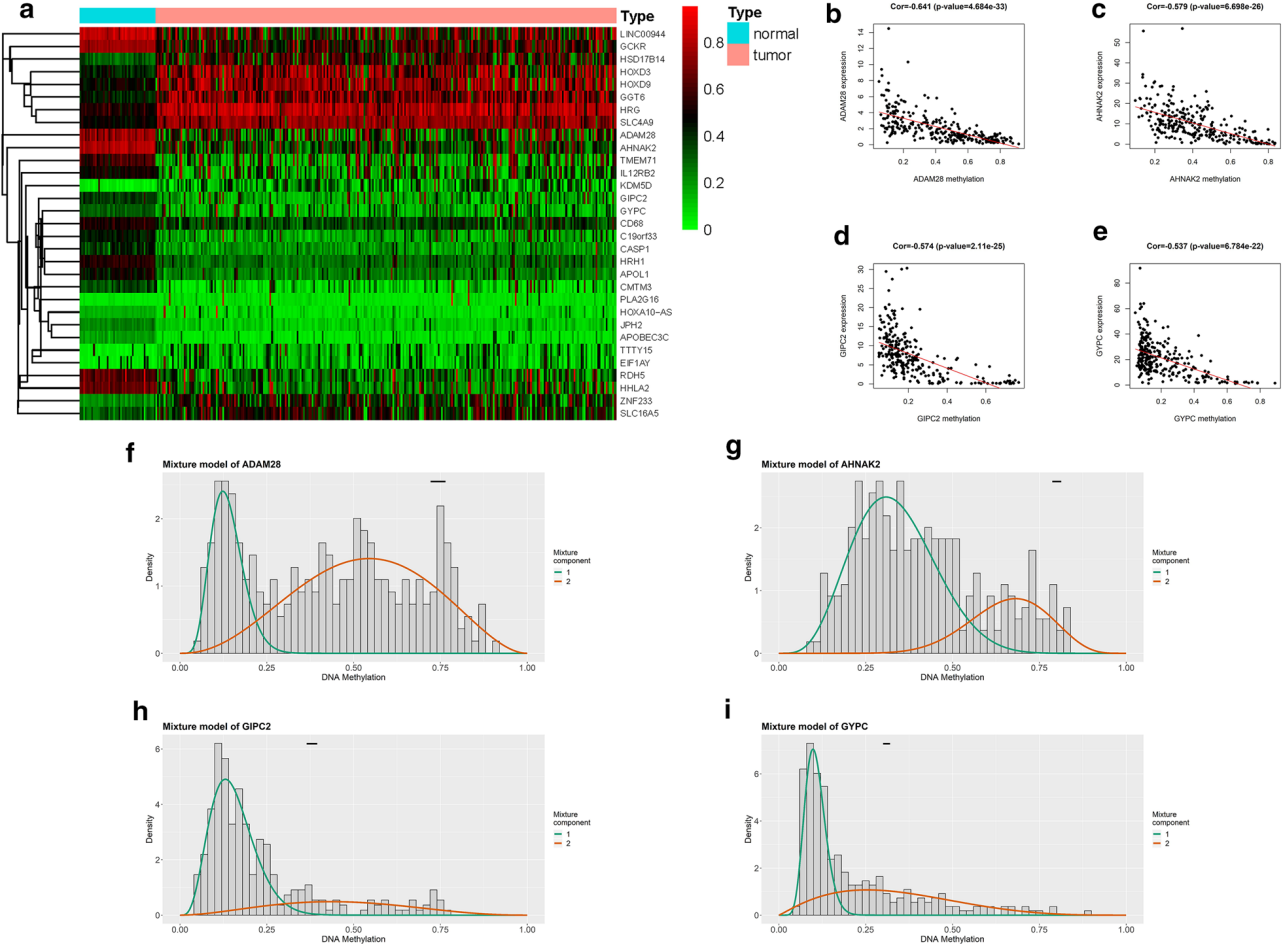
Identification of methylation-driven genes in pRCC. **a** Heat map of methylation-driven genes in pRCC. The color from green to red shows a trend from hypomethylation to hypermethylation. **b–e** The correlation between methylation and gene expression in methylation-driven genes. **f–i** The methylation degree when comparing cancer samples to normal samples in pRCC. The histogram represents the distribution of methylation in tumor samples. The black line above the figure demonstrates the distribution of methylation levels in normal samples

In the present study, the methylation data analyzed were collected from 321 samples, including 276 tumor samples and 45 non-tumor samples. Gene expression data were obtained from 321 samples, including 289 tumor samples and 32 non-tumor samples. Both DEGs and DMGs were analyzed using the limma package of R software (Additional file 1). The data were then merged using the MethylMix package. Altogether, 31 methylation-driven genes were obtained (Table 1, Fig. 1). Figure 1 shows that four methylation-driven genes (ADAM28, AHNAK2, GIPC2, and GYPC) have significant negative correlations between methylation and gene expression levels.

**Table 1:** Methylation-driven genes

Gene	Normal mean	Tumor mean	LogFC	P value	Cor	Cor P value
AHNAK2	0.80029	0.416044	− 0.94379	5.37E − 24	− 0.57876	6.70E − 26
HRH1	0.544689	0.294975	− 0.88484	5.82E − 24	− 0.38988	2.22E − 11
HRG	0.522776	0.789381	0.59453	2.59E − 23	− 0.33909	8.47E − 09
JPH2	0.235062	0.112414	−1.06422	2.89E − 22	− 0.37387	1.62E − 10
SLC4A9	0.479872	0.713725	0.572717	6.78E − 22	− 0.35973	8.57E − 10
APOL1	0.477775	0.267652	− 0.83597	2.32E − 21	− 0.34269	5.75E − 09
CASP1	0.396953	0.210789	− 0.91317	2.67E − 20	− 0.49442	2.69E − 18
GGT6	0.426959	0.665606	0.64057	3.09E − 20	− 0.33194	1.80E − 08
RDH5	0.638209	0.320082	− 0.99559	3.66E − 20	− 0.42865	1.13E − 13
CD68	0.530169	0.367352	− 0.52929	3.72E − 20	− 0.35661	1.22E − 09
GCKR	0.765666	0.540347	− 0.50283	6.32E − 19	− 0.35733	1.13E − 09
APOBEC3C	0.185781	0.082609	− 1.16923	1.06E − 18	− 0.60805	4.30E − 29
LINC00944	0.831985	0.530775	− 0.64846	1.06E − 18	− 0.46232	6.47E − 16
C19orf33	0.437952	0.215921	− 1.02027	4.00E − 18	− 0.3937	1.36E − 11
CMTM3	0.342488	0.143439	− 1.25562	6.58E − 17	− 0.39415	1.28E − 11
ADAM28	0.74307	0.42393	− 0.80967	2.72E − 16	− 0.64063	4.68E − 33
HHLA2	0.662209	0.341074	− 0.9572	5.35E − 16	− 0.47839	4.46E − 17
HOXD9	0.480952	0.68171	0.503265	7.76E − 16	− 0.42839	1.18E − 13
HOXA10-AS	0.15125	0.098828	− 0.61395	1.25E − 15	− 0.45093	3.96E − 15
TTTY15	0.063782	0.192718	1.595264	1.47E − 14	− 0.38672	3.31E − 11
ZNF233	0.221283	0.434761	0.974332	8.90E − 14	− 0.4618	7.04E − 16
HOXD3	0.419182	0.655494	0.645008	9.57E − 14	− 0.33546	1.25E − 08
GIPC2	0.379961	0.229483	− 0.72747	1.00E − 13	− 0.5739	2.11E − 25
KDM5D	0.05324	0.237807	2.159199	1.34E − 13	− 0.32883	2.49E − 08
HSD17B14	0.303245	0.525014	0.791874	7.49E − 13	− 0.65209	1.45E − 34
SLC16A5	0.305615	0.45648	0.578838	1.11E − 10	− 0.61938	2.03E − 30
TMEM71	0.591421	0.308036	− 0.94109	1.60E − 10	− 0.65122	1.89E − 34
GYPC	0.310257	0.213077	− 0.54209	9.91E − 10	− 0.53728	6.78E − 22
IL12RB2	0.504344	0.315692	− 0.67589	1.25E − 08	− 0.50024	9.33E − 19
EIF1AY	0.108578	0.182552	0.74958	1.90E − 06	− 0.40683	2.40E − 12
PLA2G16	0.053717	0.088753	0.724419	3.68E − 06	− 0.36133	7.13E − 10

### Functional enrichment, pathway and genetic alteration analysis of methylation-driven genes

**Fig. 2:**
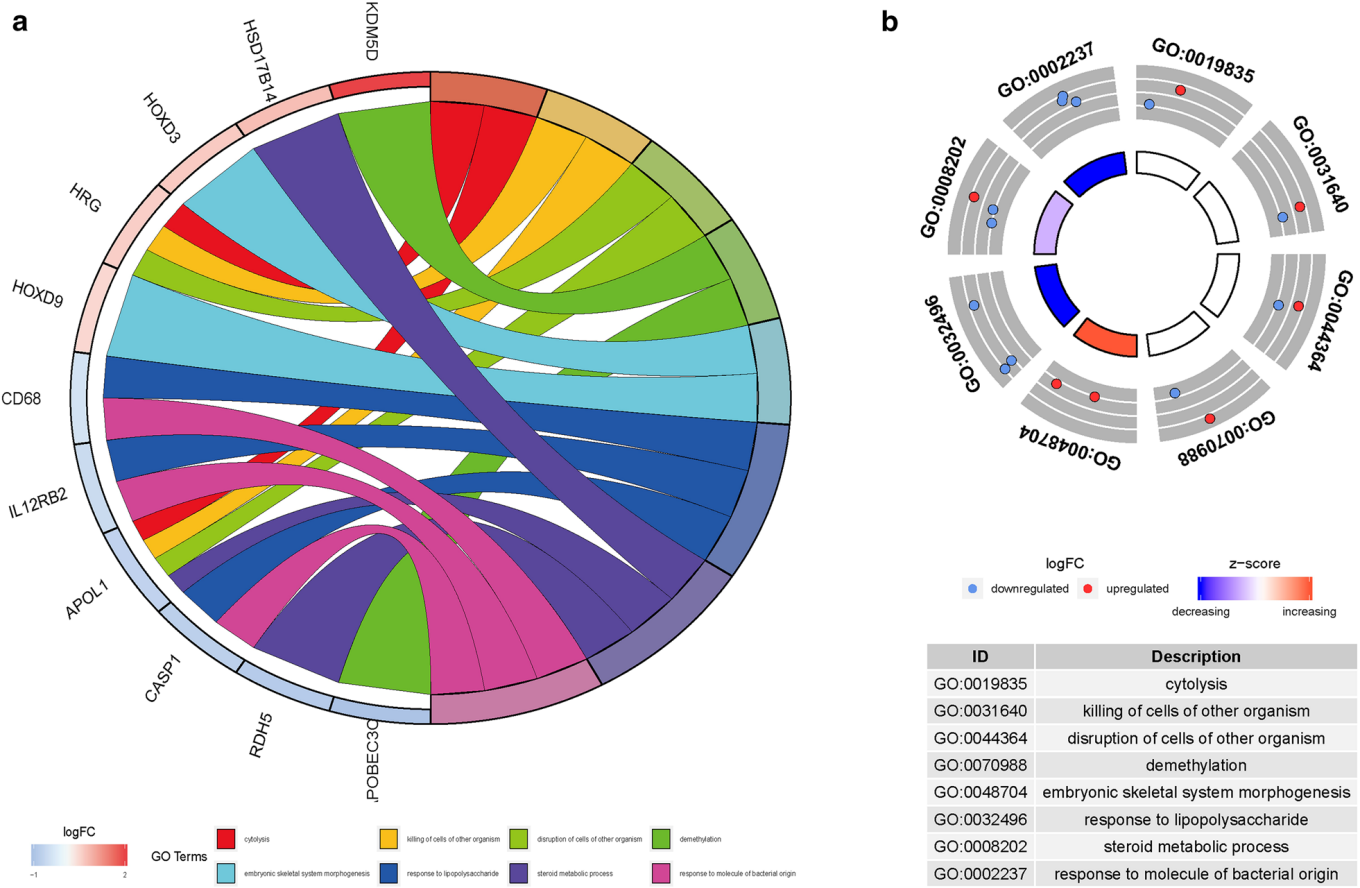
Functional enrichment analysis of methylation‑driven genes in pRCC. **a** The circle indicates the correlation between the top 11 methylation‑driven genes and their gene ontology terms. **b** The outer circle represents the expression (logFC) of methylation‑driven genes in each enriched GO (gene ontology) term: red dots on each GO term indicate upregulated methylation‑driven genes and blue dots indicate downregulated methylation‑driven genes. The inner circle indicates the significance of GO terms (log10‑adjusted P values)

**Fig. 3:**
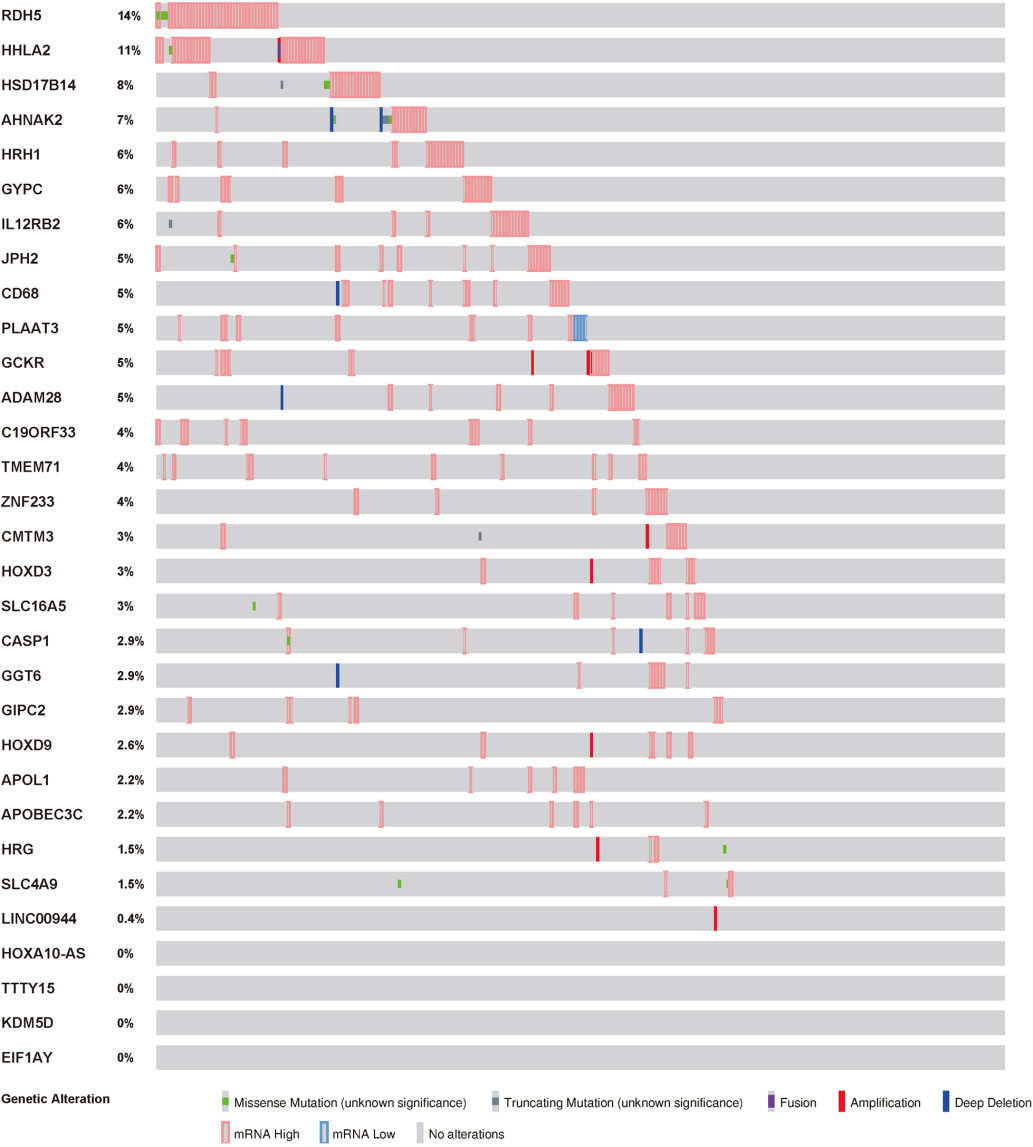
The genetic alteration of 31 genes in pRCC patients using the cBioPortal database

GO enrichment analysis revealed that methylation-driven genes were mainly enriched on multiple BP, MF, and CC terms (Fig. 2, Table 2). According to the P value from low to high, the top BP terms were cytolysis, the killing of cells of other organism, disruption of cells of other organism, and demethylation. In MF, the top terms were steroid dehydrogenase activity, anion transmembrane transporter activity, and oxidoreductase activity. In CC, Z disc, I band, and blood microparticle were the top-ranking terms. Pathway enrichment analysis showed that genes were closely related to the taurine and hypotaurine metabolism pathway and Salmonella infection pathway (Table 3). The cBioPortal tool was used to analyze 31 methylated genes for genetic alterations. As shown in Fig. 3, RDH5 and HHLA2 were the most common altered genes. Of the 274 samples, 186 (68%) samples had genetic alterations, and the changes were mainly mRNA upregulation.

**Table 2:** Functional enrichment analysis of methylation-driven genes associated with papillary renal cell carcinoma

Category	ID	Term	P value
BP	GO:0019835	Cytolysis	0.001519
BP	GO:0031640	The killing of cells of other organism	0.00361
BP	GO:0044364	Disruption of cells of other organism	0.00361
BP	GO:0070988	Demethylation	0.004578
BP	GO:0048704	Embryonic skeletal system morphogenesis	0.007946
CC	GO:0030018	Z disc	0.013994
CC	GO:0031674	I band	0.016283
CC	GO:0072562	Blood microparticle	0.017152
CC	GO:0061702	Inflammasome complex	0.019008
CC	GO:0043034	Costamere	0.025712
MF	GO:0033764	Steroid dehydrogenase activity, acting on the CH-OH group of donors, NAD or NADP as acceptor	0.0007
MF	GO:0016229	Steroid dehydrogenase activity	0.00102
MF	GO:0008509	Anion transmembrane transporter activity	0.00949
MF	GO:0016616	Oxidoreductase activity, acting on the CH-OH group of donors, NAD or NADP as acceptor	0.011235
MF	GO:0016614	Oxidoreductase activity, acting on CH-OH group of donors	0.012911

If there were more than five terms in this category, the first five terms were selected based on the P value

**Table 3:** Pathway analysis of methylation-driven genes associated with papillary renal cell carcinoma

ID	Pathway	P value
hsa00430	Taurine and hypotaurine metabolism	0.016365
hsa05132	Salmonella infection	0.039306

### Prognostic model construction and survival analysis

**Fig. 4:**
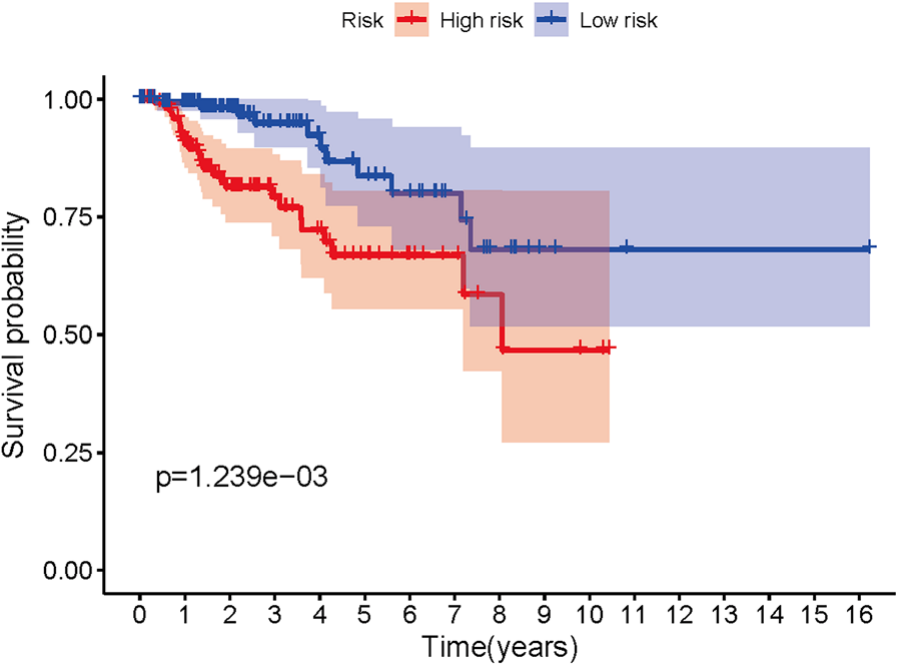
Kaplan–Meier curve analysis for OS (overall survival) of pRCC patients using the 8 genes signatures

**Fig. 5:**
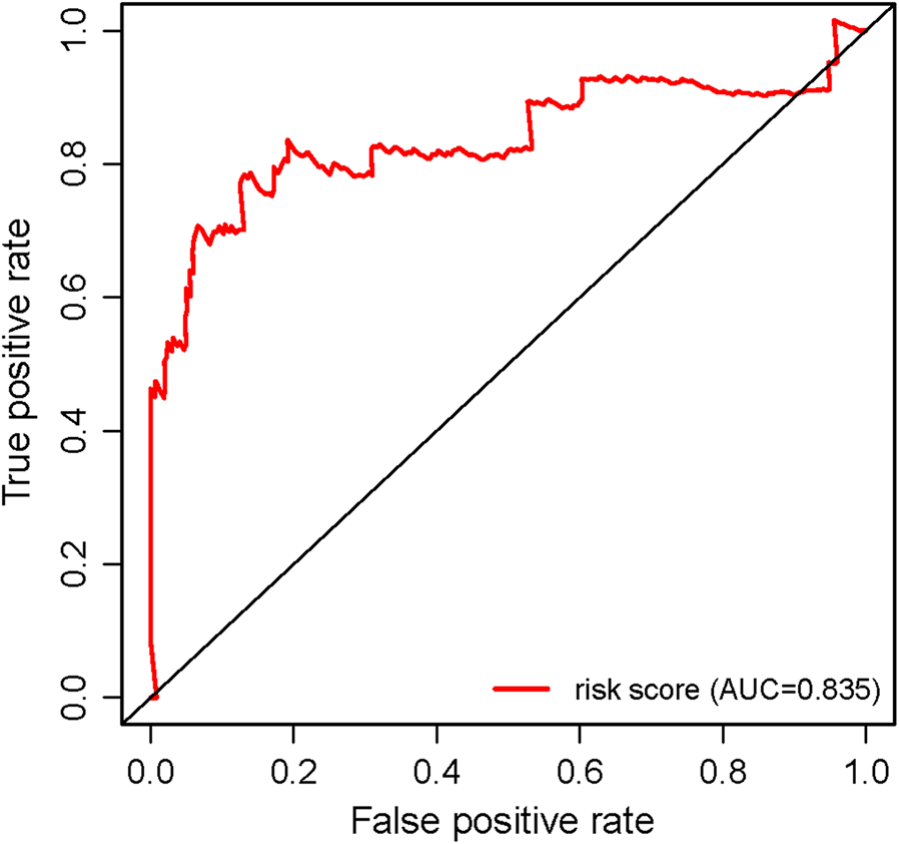
Time-dependent ROC curves analysis for 3-year survival prediction by methylation-driven genes

**Fig. 6:**
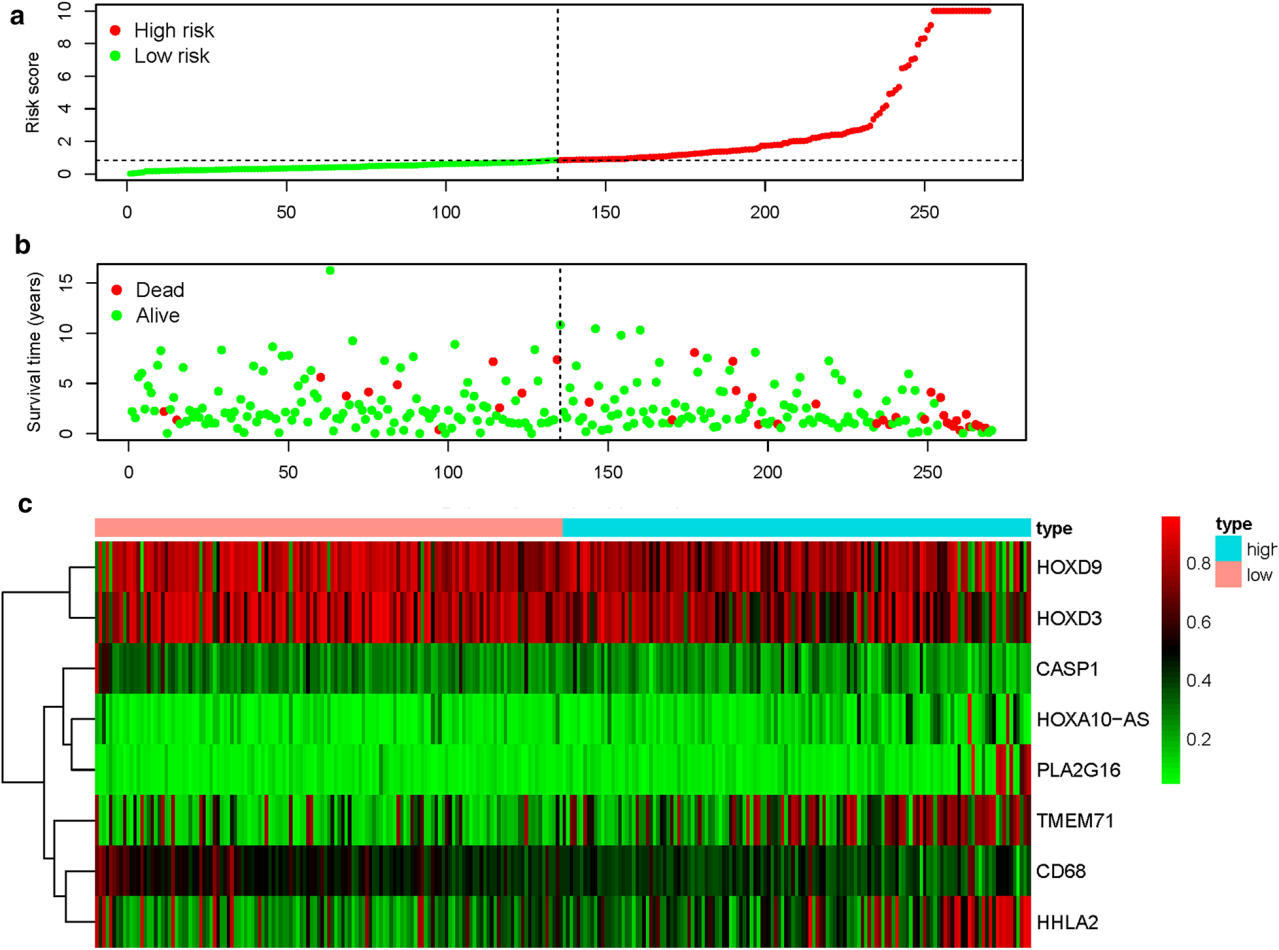
Methylation-driven genes risk score analysis of pRCC. **a** Rank of risk score and distribution of groups. **b** The survival status of pRCC patients in different groups. **c** Heatmap of methylation profiles of the 8 key methylation-driven genes. The color from green to red shows an increasing trend from low levels to high levels

**Fig. 7:**
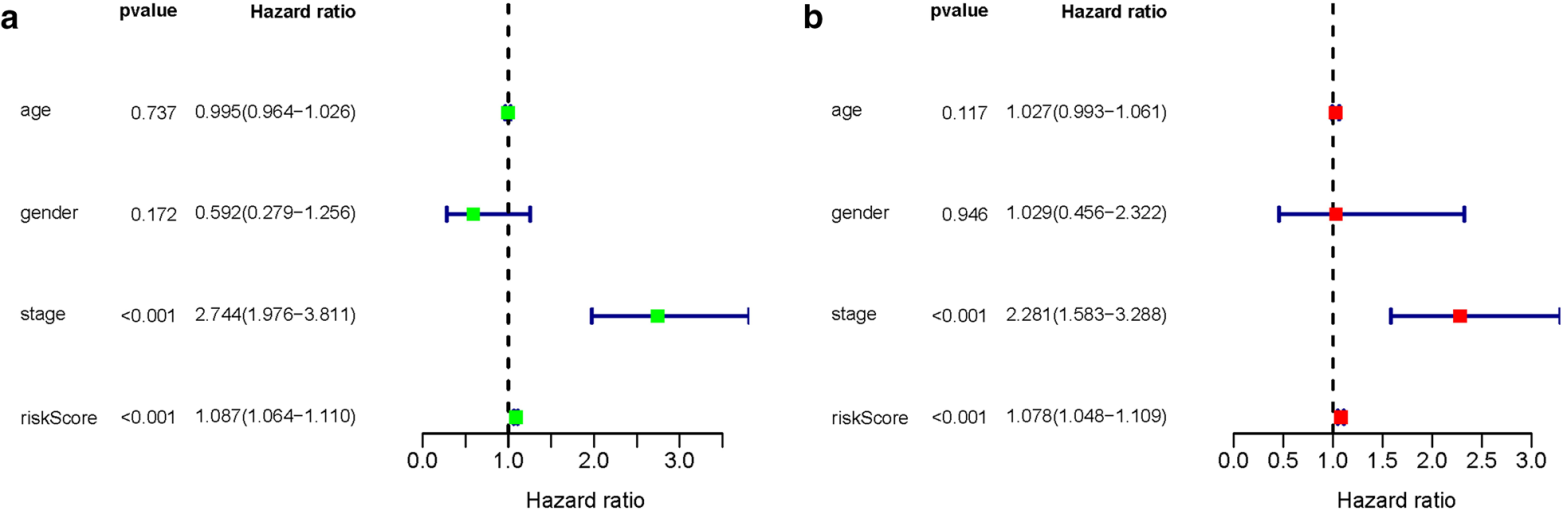
Univariate and multivariate analyses of overall survival in pRCC patients of TCGA **a** Univariate analysis, **b** Multivariate analysis

**Fig. 8:**
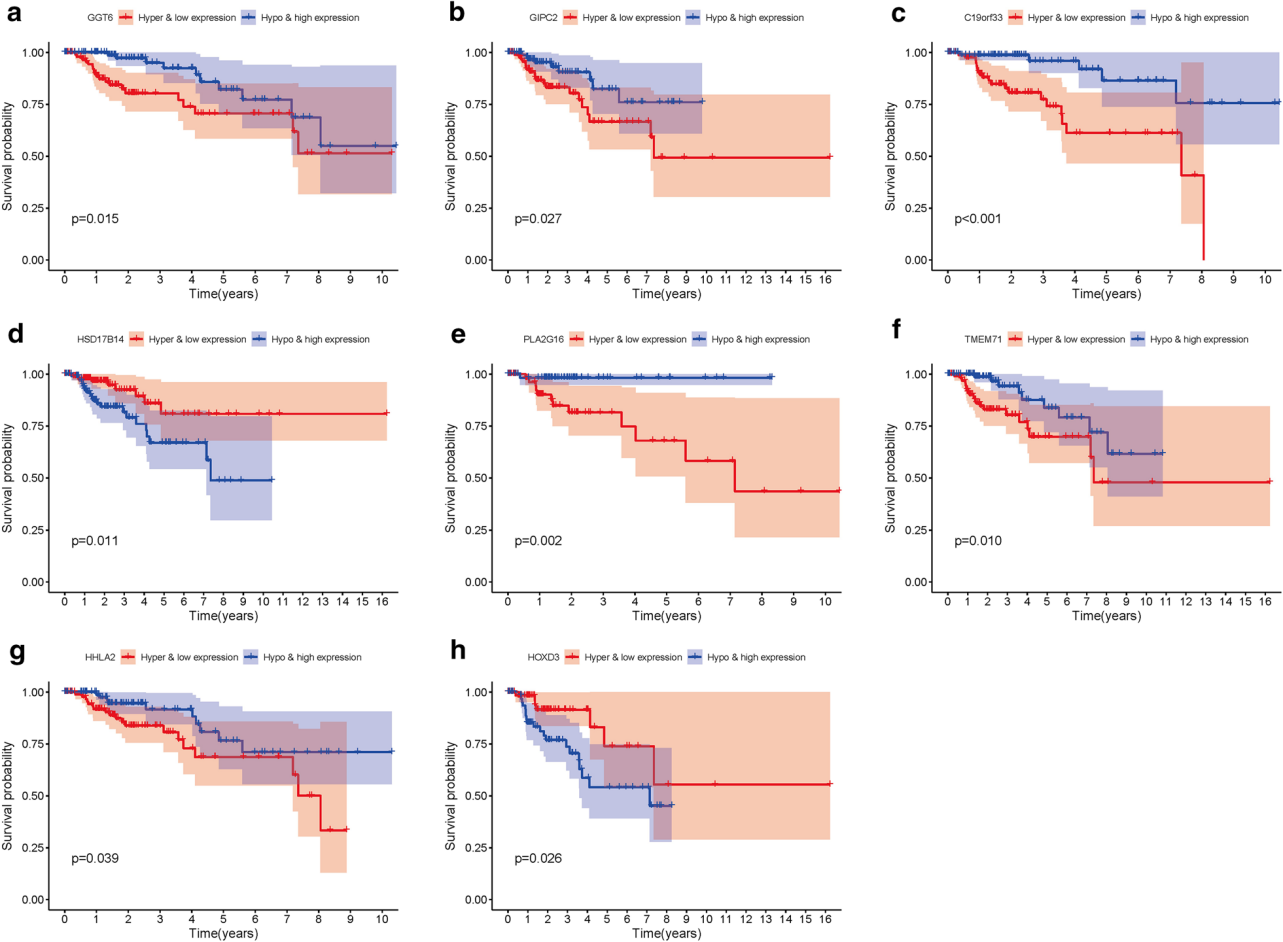
Kaplan–Meier survival curves for the joint survival analysis

A total of 14 genes related to pRCC prognosis were detected using univariate Cox regression analysis. Next, we selected the linear combination of the methylation of the eight genes to construct the predictive model via multivariate Cox regression analysis. The relative coefficients weighted in the multivariate Cox regression were as follows: survival risk score = (−5.8122) × methylation value of CASP1 + (−7.457) × methylation value of CD68 + 1.9948 × methylation value of HHLA2 + 2.988 × methylation value of HOXD9 + 4.6693 × methylation value of HOXA10-AS + (−1.6449) × methylation value of HOXD3 + 1.4863 × methylation value of TMEM71 + 5.4692 × methylation value of PLA2G16 (Table 4). Among these genes, the methylation levels of CASP1, CD68, and HOXD3 negatively correlated with a risk score, and the remaining five positively correlated with a risk score. The Kaplan–Meier survival curve indicated a statistical difference in survival between the high and low-risk groups (Fig. 4). The ROC curve in a risk scoring model was used to evaluate its predictive performance. The area under the curve (AUC) of the prognostic risk assessment model for the eight methylation-driven genes was 0.835 at 3 years of overall survival (Fig. 5), suggesting the model is able to predict the prognostic risk in pRCC patients. Meanwhile, we also recorded patient’s risk score, survival status, and methylation of eight genes (Fig. 6). After adjusting parameters, including age, gender, and pathological stage, the risk score was found to be an independent predictor using multivariate Cox regression analysis (Fig. 7). Joint survival analysis of gene expression and methylation showed that methylation and expression of C19orf33, GGT6, GIPC2, HHLA2, HOXD3, HSD17B14, PLA2G16, and TMEM71 were significantly correlated with the prognosis of pRCC patients (Fig. 8). Hypermethylation and low expression of HOXD3 and HSD17B14 indicate high survival, while hypermethylation and low expression of C19orf33, GGT6, GIPC2, HHLA2, PLA2G16, and TMEM71 suggests low survival.

**Table 4:** Multivariate Cox regression analysis of 8 genes associated with overall survival in papillary renal cell carcinoma patients

ID	Coef	HR (95%CI)	P value
CASP1	− 5.8122	0.002991 (9.19E − 06 − 0.973672)	0.048953
CD68	− 7.45701	0.000577 (1.00E − 05 − 0.033249)	0.000311
HHLA2	1.994774	7.350543 (0.931728 − 57.98953)	0.058376
HOXD9	2.988006	19.84608 (1.03704 − 379.7992)	0.047243
HOXA10-AS	4.66935	106.6284 (4.150071 − 2739.618)	0.004814
HOXD3	− 1.64487	0.193038 (0.030944 − 1.204217)	0.078236
TMEM71	1.486315	4.420773 (1.056385 −18.50011)	0.041844
PLA2G16	5.469168	237.2627 (19.32855 − 2912.458)	1.91E − 05

## Discussion

The molecular mechanism of pRCC tumorigenesis remains unclear. In-depth research on the molecular pathogenesis of pRCC, as well as early detection of prognostic markers and specific driven genes for the disease, is of great significance for improving patient prognosis and developing new drugs. Epigenetics refers to the phenomenon of heritable and reversible changes in gene expression caused by non-DNA sequence changes. Increasing studies show that epigenetic modification closely relates to the occurrence and development of cancer [26]. Because epigenetic changes are reversible, they have more potential as new therapeutic targets than mutations in the DNA sequence. Multiple studies demonstrate the correlation between epigenetic changes and tumor prognosis, which indicates that they may be a factor in predicting tumor prognosis. For instance, Zhang et al. [27] found that the reduced expression of ZNF671 caused by hypermethylation is associated with poor prognosis in multiple solid tumors. Gao et al. [28] established a predictive risk model assessing the prognosis of patients with lung squamous cell carcinoma by studying the abnormal methylation sites of key genes with a poor prognosis. Therefore, it is necessary to explore the relationship between pRCC-related epigenetic changes and the patient’s prognosis.

In this study, we found DEGs and DMGs between tumor samples and adjacent non-tumor samples, and further identified prognostic biomarkers associated with methylation-driven genes. We analyzed methylation and gene expression using the MethylMix package and detected 31 methylation driven-genes. GO analysis indicates that the methylation-driven genes in pRCC are mainly enriched in steroid dehydrogenase activity, anion transmembrane transporter activity, and oxidoreductase activity in MF. In CC, these genes are enriched on the Z disc and I band. In addition, in BP, enrichment is primarily seen in the regulation of cytolysis and demethylation. Pathway enrichment shows that methylation-driven genes closely relate to taurine and hypotaurine metabolism. These terms reflect gene-to-gene interactions at the functional level and indicate that gene dysfunction may be due to abnormal DNA methylation in different samples.

To further investigate the relationship between methylation-driven genes and patients with pRCC, we analyzed the relationship between abnormal DNA methylation and patient’s survival via the survival R package. Eight candidate genes were identified from 31 methylation-driven genes and a predictive risk model was constructed. Using this risk model, we successfully divided the pRCC samples into high-risk and low-risk groups. As shown in Fig. 4, the 4-year survival rate in the high-survival group was nearly 90%, while that of the low-survival group was about 70%. Survival after 8 years in the high-survival group was about 70%, while survival in the low-survival group was only about 40%. Survival analysis showed a significant difference in overall survival between the two groups. The results show that a risk model consisting of eight methylation-driven genes can effectively predict the prognosis of patients with pRCC. Furthermore, the results also showed that the AUC of the ROC curve of eight gene signatures predicting 3-year survival was 0.835. Multivariate Cox analysis showed that the model’s risk score might be used as an independent prognostic factor for pRCC. We found good performance in the pRCC patients’ survival prediction using the risk assessment model constructed by the eight gene signatures, but further research is necessary to validate these findings. These methylation-driven genes can serve as effective biomarkers or drug targets for early diagnosis and prognosis of pRCC patients. However, the influence of abnormal methylation data on patient survival is not comprehensive. Therefore, we combined methylation-driven genes and corresponding gene expression data with patient survival for an integrated analysis. The results found that C19orf33, GGT6, GIPC2, HHLA2, HOXD3, HSD17B14, PLA2G16, and TMEM71 significantly correlated with prognosis. Hypermethylation and low expression of HOXD3 and HSD17B14 led to high survival, while hypermethylation and low expression of C19orf33, GGT6, GIPC2, HHLA2, PLA2G16, and TMEM71 led to low survival.

HHLA2 is involved in the regulation of T cells [29, 30]. Byers et al. [31] found that HHLA2 expression is downregulated or deleted in pancreatic cancer tissues, which may contribute to the immune escape of pancreatic cancer. In a colorectal cancer study [32], HHLA2 expression in cancer tissues was higher than in adjacent tissues, and the expression level was significantly associated with the depth of invasion and CD8 + T cell infiltration status and could predict high mortality rates. This may be related to the specificity of different tumors. Our study found that the hypermethylation of HHLA2 leads to a poor prognosis. We speculate that the low expression caused by HHLA2 hypermethylation mediates the immune escape of pRCC.

HOXD3 mainly plays a role in regulating cell proliferation and differentiation in the body [33, 34]. Other studies observed that HOXD3 overexpression serves as an independent risk factor for poor prognosis of breast cancer [35]. Joint survival analysis showed that overexpression of HOXD3 was associated with poor prognosis in patients with pRCC. This may occur because HOXD3 can regulate the downstream transfer of related molecules and adhesion molecules, such as integrin β3, urokinase plasminogen activator, and matrix metalloproteinases, and subsequently induce tumor angiogenesis, regulate tumor cell apoptosis, and enhance tumor cell invasion and metastasis [36]. PLA2G16 is a tumor suppressor gene that can induce programmed cell death [37] and inhibit cell migration and invasion [38]. Jarrard et al. [39] observed that PLA2G16 methylation in urine and prostate tissues can detect the presence of prostate cancer, indicating that downregulation of the PLA2G16 gene may play an important role in multifocal prostate carcinogenesis. In our study, we observed that a high level of PLA2G16 gene methylation was associated with low survival in patients with pRCC.

A study by Wang et al. [40] showed that TMEM71 acts as an oncogene in glioblastoma multiforme and is associated with an immune response. Joint survival analysis observed that hypermethylation and low expression of TMEM71 were associated with poor prognosis in patients with pRCC. CASP1 belongs to the cysteinyl aspartate-specific proteases family and plays an important role in the processes of inflammatory response [41] and cell pyroptosis [42]. In the risk model, the methylation level of CASP1 was negatively correlated with the risk score, indicating that the high expression of CASP1 was a high-risk factor, which might be related to CASP1’s activation of IL-1β, inducing inflammation and immunosuppression to promote tumor growth and metastasis [43]. CD68 is a widely used macrophage marker that can be used to assess the extent of macrophage infiltration in tumor tissue [44]. The risk model showed that the expression level of CD68 was positively correlated with the risk score, which was consistent with the current literature reports [45], suggesting that tumor-associated macrophages might be involved in tumor progression in pRCC.

Currently, there are no studies on the role of HOXA10-AS in pRCC. Some studies have shown that high expression of HOXA10-AS in lung adenocarcinoma [46] and glioma [47] can promote tumor progression. According to the risk model, hypermethylation and low expression of HOXA10-AS can increase patient survival risk, which may be related to the heterogeneity of different types of tumors. Kishibuchi et al. [48] found that promoter methylation of HOXD9 was significantly higher in thymic carcinoma than in thymoma and the thymus, and relapse‑free survival was significantly worse in tumors with a higher DNA methylation of HOXD9 in all of the thymic epithelial tumors. Similar to the results of this study, hypermethylation of HOXD9 was positively correlated with high risk score. However, the specific role of HOXD9 methylation in tumor progression needs to be further studied. These genes may become new targets for treating and improving the prognosis of patients with pRCC.

Currently, few studies on pRCC abnormal methylation genes have been reported. Compared with previous studies, we conducted a more comprehensive analysis of the methylation-driven genes in pRCC using the MethylMix algorithm. In addition, we analyzed the correlation between abnormal methylation sites and gene expression, providing accurate targets for further experimental verification. Our research subjects were retrieved from the TCGA database, which is an important tool for analyzing prognostic biomarkers. Although we have investigated the relationship between epigenetic changes and pRCC, the prediction of gene signatures for prognosis still needs to be verified by molecular biology experiments based on clinical samples in the future, and its specific molecular mechanism in pRCC requires further testing.

## Conclusion

Using the transcriptome profile data and genomic methylation data of pRCC patients in the TCGA database, this study obtained 31 pRCC-related methylation-driven genes and constructed a prognostic survival model with eight methylation-driven genes (CASP1, CD68, HOXD3, HHLA2, HOXD9, HOXA10-AS, TMEM71, and PLA2G16). These eight genes may be involved in various processes related to pRCC, such as immune escape, inflammatory response, apoptosis and cell migration. Additionally, these genes have the potential to be markers or drug targets for early diagnosis and protocol evaluation of pRCC. Although further experimental verification is needed, our findings provide essential bioinformatics and related theoretical foundations for future pRCC studies.

## Supplementary information

**Additional file 1:** Results of the limma package.

## Data Availability

The authors declare that the data supporting the findings of this study are available in the TCGA database. (https://portal.gdc.cancer.gov/).

## Abbreviations

TCGA: The Cancer Genome Atlas
RCC: Renal cell carcinoma
pRCC: Papillary renal cell carcinoma
ccRCC: Clear cell renal cell carcinoma
DEG: Differentially expressed gene
DMG: Differentially methylated gene
MDG: Methylation-driven gene
ROC: Receiver operating characteristic
AUC: Area under the curve
GO: Gene ontology
BP: Biological process
MF: Molecular function
CC: Cellular component
